# When sex meets age: Family physicians’ perspectives about sexual dysfunction among older men and women: A qualitative study from Israel

**DOI:** 10.1080/13814788.2019.1580263

**Published:** 2019-03-08

**Authors:** Inbar Levkovich, Ateret Gewirtz-Meydan, Khaled Karkabi, Liat Ayalon

**Affiliations:** a The Division of Family Medicine, The Ruth & Bruce Rappaport Faculty of Medicine, Technion-Israel Institute of Technology, Haifa, Israel;; b The Louis and Gaby Weisfeld School of Social Work, Bar-Ilan University, Ramat Gan, Israel;; c Sex and Couple Therapy Unit, Meir Medical Center, Kfar Saba, Israel;; d Department of Family Medicine, The Ruth & Bruce Rappaport Faculty of Medicine, Clalit Health Services, Haifa and Western Galilee District, Technion-Israel Institute of Technology, Haifa, Israel

**Keywords:** Aging, older adults, primary care, sexual dysfunction, sexuality

## Abstract

**Background:** Gender differences in relation to sexual functioning among older adults have received very little research attention, although the ageing process is likely to be characterized by difficulties in sexual functioning among both women and men.

**Objectives:** The purpose of this qualitative research is to examine and understand the perceptions of family physicians, and the differences in their attitudes regarding male and female patients.

**Method:** Qualitative interviews with family physicians were conducted between August 2017 and December 2017. Sixteen family physicians participated in the study, aged 36–64; most were born in Israel and half of them were women. Twelve physicians were Jewish, two were Christian and two were Muslim. Nine work in rural practices and seven work in urban practices. We used in-depth, semi-structured, face-to-face interviews. The interviews were recorded, transcribed and analysed by three researchers using content analysis.

**Results:** The analysis of the interviews revealed two main themes: (1) Differences as perceived by family physicians: men are perceived as being interested in engaging in full sexual relations, including penetration, while among women, the main need is focused on the relationship and intimacy. (2) Gender differences regarding seeking a solution through the family physician. Family physicians reported that most of the patients who seek solutions regarding sexual dysfunction in old age are men with impotence problems. Family physicians perceived that women seek out solutions less frequently, some because they are afraid their relationship will suffer if they do not continue having sex with their partners.

**Conclusion:** Men and women were seen as having different motivations for engaging in sex and different needs from physicians.

KEY MESSAGES

- Family physicians perspective that men interested in engaging in full sexual relations, while women main need is focused on relationship and intimacy.
- Family physicians perspective that men's problems simple and easily solved, whilst the women's problems were more complex.
- Physician knowledge should improve patient counseling.

## Introduction

Sexual activity and sexual satisfaction continue to play an important role in the lives of older adults, both women and men [1–3]. In a study that examined sexual function among older adults, 58% of the men and 51% of the women reported having sexual relations during the past year. Yet, the ageing process is likely to be accompanied by a difficulty to engage in sexual activity [4–6]. Among older adults with active sex lives, half of the men and women report at least one problem concerning sexual function [7].

Among men, the most common sexual problem is erectile dysfunction (ED) 20–40% for men in their 60s and 50–70% for men aged 70 and above indicate suffering from ED [6,8,9]. Among older women, sexual function diagnoses are more complex and there is a certain vagueness about the diagnoses [4]. The most common sexual dysfunction reported by women aged 57–85 is reduced libido levels, 43%; vaginal lubrication difficulties, 39% [8]; and inability to climax, 34%. The period of menopause, characterized by complex hormonal changes, includes a decrease in testosterone and DHEA (dehydroepiandrosterone), which affect women’s sexual function [3].

Despite the existing knowledge about the importance older people give to staying sexually active [9], physicians do not perceive their patients’ sexuality as part of their field of expertise; they report a lack of knowledge and a lack of confidence regarding the subject [10,11]. Moreover, higher levels of ageist attitudes were found to be directed towards older women compared to older men [12]. Older men and women are often afraid to involve physicians in their sexual problems and therefore choose to deal with these problems alone or to ignore them [13–18]. When family physicians raise sexual issues, it usually occurs with older men, rather than with older women [10], despite studies, which indicate a higher sexual dysfunction rate among older women [3].

Family physicians play a significant role in treating sexual functioning among older adults. In light of the apparent differences in the literature about the attention given to older men in comparison with older women in diagnosing and treating sexual functioning difficulties [4,7,19], the goal of the qualitative study was to examine the attitudes of family physicians and understand their perspective and the differences in their attitudes towards their male and female patients.

## Method

### Research population

This qualitative study was supported by a grant from the Israel National Institute for Health (Policy Research No. א/2016/16.). The study focuses on a sample of 16 family physicians in Israel. The study used a qualitative-phenomenological approach [20]. This approach attempts to obtain an in-depth understanding of the phenomenon by entering the world and experiences of the participants. The qualitative-phenomenological research approach was chosen to enable family physicians to tell their stories and give meaning to their experiences. The sample emphasizes a rich and diverse conceptualized representation for background variables and in the way it represents the family physician population in Israel. In the study, 16 family physicians participated, aged 36–64; most were born in Israel, eight women and eight men (see Table 1). Twelve of the physicians were Jewish, two were Christian and two were Muslim. Nine work in rural practices and seven in urban practices. Thirteen work in the ‘Clalit’ Healthcare Services and three in the ‘Maccabi’ Healthcare Services, the two largest healthcare services in Israel.

**Table 1.** Background data of family physicians participating in the qualitative study.

	Pseudonym	Sex	Age	Marital status	Years of experience	Place of birth
1	Hannah	Female	36	Married	9	Israel
2	Kim	Female	38	Married	7	Germany
3	Manar	Female	38	Married	11	Israel
4	Lily	Female	42	Divorced	10	Israel
5	Sofia	Female	42	Married	12	Israel
6	Emma	Female	43	Married	13	Israel
7	Jonathan	Male	45	Married	15	Israel
8	William	Male	45	Married	16	Israel
9	Olivia	Female	46	Divorced	16	Israel
10	Ava	Female	46	Married	16	Israel
11	Jacob	Male	47	Married	18	Israel
12	James	Male	50	Married	20	Ukraine
13	Daniel	Male	52	Married	23	Israel
14	Ryan	Male	54	Married	23	Israel
15	Michael	Male	61	Married	21	Israel
16	Mohammed	Male	64	Married	38	Israel

### Ethics

The Hospital Ethics Committee approved study No. 0262-16-MMC. The researchers identified the participants and requested their written consent to participate in the study.

### Research tools and instruments

A psychotherapist trained in conducting qualitative-phenomenological interviews conducted the individual in-depth, semi-structured interviews. The interview was used as an instrument to help us learn about and examine the participants’ experiences. An interview guide was developed, drawing on the literature. The interview was conducted based on an interview guide, which included significant key areas, but was flexible enough to allow for the development of a dialogue between the interviewer and the interviewee and meaningful self-expression [21].

The interview guide included several questions: ‘Tell me about sexual function in old age: What are your reactions (thoughts, feelings, behaviours);’ ‘When do patients consult with you about sexual function in old age?;’ “How do you handle sexual dysfunction in older adults?;’ ‘What are the advantages and disadvantages of the treatment given to alleviate sexual dysfunction?;’ and ‘How do sexual difficulties among older adults differ from those of younger adults?’ The interviewer encouraged physicians to narrate their experiences in their own words [21].

### Research procedure

The Hospital Ethics Committee approved study No. 0262-16-MMC. A personal email was sent to 20 family physicians, to ensure that a variety of background characteristics would be included (gender, age, religion, years in the profession, clinic type). Sixteen family physicians agreed to participate in the study. These physicians were interviewed, yielding an 80% response rate. The researchers selected the participants and requested their agreement to participate in the study. The participants received a comprehensive explanation about the study and they were interviewed in their homes or work clinic, according to their preference. The interview lasted for one hour and it was conducted in Hebrew. Each interview was recorded and then transcribed. Data collection proceeded until ‘theoretical saturation’ had been reached (i.e., additional interviews yielded no new material for analysis).

### Data analysis

The interviews were analysed using content analysis, to identify and code central themes and patterns. In the first stage, all of the interviews were read and analysed line by line. Then, the interview’s main themes were identified, along with sub-categories. In the next stage, the researchers looked for related themes and the sub-categories were grouped into secondary categories. In the last step, we identified the study’s central themes [21].

## Results

An analysis of the qualitative interviews revealed two main themes:

1. ‘Family physicians’ perceptions of patients’ sexual goals.’ This theme dealt with family physicians’ attitudes towards the different needs of men and women. Attitudinal differences are described: men are perceived as wanting to engage in full sexual relations including penetration, and difficulties in sexual function were perceived as influencing their self-confidence. Physicians perceive women’s needs as being focused on the relationship and intimacy.
2. The second theme, ‘Family physicians’ perceptions of how patients deal with the problematic situation’ dealt with gender differences regarding seeking help from a family physician because of sexual function problems. Family physicians said that most of the sexual function problems reported among the older population come from men experiencing impotence. Women seek out help less frequently and their direct complaints often relate to gynaecological symptoms rather than sexual function problems. Family physicians perceived men’s problems as common, and easily solvable through medication. Women’s complaints were perceived as more complex, requiring a solution involving both medication and emotional support.

Below is a detailed account of the two themes supported by direct quotes from the interviews.

Theme one. ‘Family physicians’ perceptions of patients’ sexual goals’

In this study, family physicians described how senior men strive to maintain an active sex life, including full penetration. Therefore, a physiological decline is often observed in their ability to engage in sexual relations, despite their motivation to have sex, and their interest in maintaining high libido levels. Because of sexual difficulties, men reported reduced confidence levels and lower quality of life to their physicians. This decline in their feelings of manliness and self-confidence often leads to disappointment and frustration:

*Among men, I think their ability often diminishes, but the desire remains. Yes, they still have urges and look at women. Sometimes, they look outside of their own relationship*. (Kim, 38)

When elderly male patients have to deal with a medical issue, such as medication or surgery, which may harm their sexual function, they hesitate or postpone the treatment, so as not to impede their ability to perform and engage in full sexual relations. Family physicians related that, in contrast with the needs of older men, older women show a need for intimacy, emotional connection, and sexual contact that does not necessarily lead to full penetration. Women show more tolerance towards their declining libido; some even perceive sexual relations as a task they must fulfil to satisfy their husbands or so as not to disappoint him or make him angry:

*Women have fewer [sexual] urges; they want it less…“Just leave me alone, in peace. And if I really have to, then okay, let’s get it over with …”* (Lily, 42)

Theme two. ‘Family physicians’ perceptions of how patients deal with the problematic situation.’

All of the family physicians said that older men dealing with sexual function difficulties ask for help more than older women. Most of the visits to family physicians were made by men with ED, caused by illness, medication, emotional difficulties, physiological decline or a combination of these factors:

*I have a patient who is around 70 years old, a man with many background diseases, high BMI, with a history of prostate cancer…some of his sexual dysfunction is also a result of surgery he underwent to treat a prostate infection. He came to me and quite freely expressed that regarding sexual function, after the operation, he was no longer the same man. He asked for help; he is taking pills. He’s a very open-minded man.* (Sofia, 42)

Some of the patients who sought advice from their family physician had experienced sexual function problems in the past but had not sought help. They decided to seek help because of a second ‘couplehood’ in old age and the new needs that had arisen.

Family physicians described how women seek help much less frequently than men do, concerning sexual dysfunction. Women who do seek help usually do so only once, even if the problem is ongoing and, as a result, they suffer over time. When they do seek help, the majority of the time, they do not look for a direct solution to the problem, but rather relate to gynaecological problems:

*Women sometimes come with all kinds of issues, such as abdominal pains, recurring urinary tract infections…the urovaginal and the urogenital areas, in general…which always makes me ask questions. And then it turns out that yes, there is a new relationship, and it’s not comfortable or it is comfortable or this or that…they did not seek help, she did not seek help;…and it is all very uncomfortable…and difficult… Many times, I ask because I know that she is a widow or that they (the couple) separated: “What is happening? Are you in a relationship with someone now?”* (Michael, 61)

Some of the patients’ visits were described as being made ‘for the partner’: The women did not come with any complaints or symptoms of their own but were mainly worried about how the lack of sex was affecting their husbands:

*Women are much more open to the idea that the family may fall apart because of tensions, arguments…women more often initiate couples therapy and everything related to it.…women understand where the problems start—that their partner’s frustration stems from this problem—so they mainly try to solve the problem in this way.* (James, 50)

## Discussion

### Main findings

The goal of the current study was to expand the understanding of family physicians’ perceptions of men and women’s sexual function in old age and to examine their attitudes about gender differences. The study indicates that family physicians perceive men and women’s needs in a different light. While men are seen as striving to engage in full sexual relations including penetration, the women’s main need was perceived as the need for intimacy and a relationship. Family physicians perceived the men’s problems as being common and easily solvable by taking medication, and observed that women seek out solutions less frequently.

### Interpretation

This finding supports previous research, which found that men report more sexual activity and thoughts about having sex compared to women [22]. Men aged 65–74 reported that they had sex 2.7 times a month compared to women who had sex 1.8 times a month [23]. Moreover, among those individuals aged 80+, who are sexually active, 19% of the men and 32% of the women reported having sex two or more times during the past month [22]. Together with having sex, another need is closeness and intimacy with one’s spouse [24]. The study shows that some of the women were described as perceiving sex as a duty or task, something they felt obliged to do to satisfy their husbands, rather than out of personal desire.

Nevertheless, studies show a rate of 25–63% for sexual dysfunction among elderly women, a lack of oestrogen being the main reason for these problems [25,26]. It was also was found that physicians who treat women with gynaecological symptoms identify a difficulty in sexual function, but very few physicians discuss this matter with the women [27]. These findings emphasize that also among women, there are medical reasons for sexual function difficulties, but these problems receive less attention and treatment [10].

According to family physicians’ perspectives in the current study, women seek help less frequently than men do, and their direct complaints often relate to gynaecological symptoms, rather than sexual dysfunction. Some of the women seek help because they are afraid they will have problems in their relationship if they do not have sexual intercourse with their partner. In Farrell and Belza’s [15] study, which examined patients’ desire to discuss this subject with their family physician, it was found that 72% of the men said they would like their family physician to ask them about sexual function and satisfaction compared to 37% of the women. Although few women are interested in discussing sexual function with their family physicians, one-third of the women said they would like their family physician to initiate such a conversation and show interest in their situation. Hence, it is important that physicians also acknowledge sexual issues with women.

The physicians’ responses showed a gender difference. Family physicians’ perceived the men’s problems as simple and easily solved using medication, while the women’s problems were perceived as more complex, requiring a solution including both medication as well as emotional support. This is in spite of the fact that other studies indicate that men’s sexual function is related to their psychological state [10,15,28–30]. The physicians’ perspectives reflect the patients’ attitudes; there is a clear public preference for treatment in the form of medication. According to an international survey, which included 12 563 people, it was found that only 7% of the people who reported suffering from ED took medication for it, but 74% claimed they were interested in taking such medication [28]. While most men find various medical solutions, the variety of solutions offered to women is narrower, which is possibly why physicians ignore them, and their emotional concerns receive more emphasis.

### Implications

The current study may contribute to improving family physicians’ approach to the subject of sexual functioning among older adults, both men and women. Sexual problems are frequent among older adults, but these problems are infrequently discussed with physicians. Physicians’ increased knowledge and attitudes about sexuality among older adults should improve patient counselling, as well as the ability to identify clinically sexual dysfunction. It is important that medical schools and medical programs specializing in family medicine will include a training program about sexuality in old age. This specialization program can assist family physicians and deepen their understanding of their attitudes when dealing with older men and women, and the difficulties and dilemmas they experience.

### Strengths and limitations

The study is qualitative, to allow an in-depth understanding of the experience and attitudes of family physicians, but its generalizability is limited and the number of participants was small. Furthermore, the physicians were asked retrospectively about their work with older patients, rather than in real time. The physicians described a heterosexual patient population; thus, a more diverse population regarding sexual identification was not represented. It is recommended that future studies will also examine patients’ attitudes, both men and women, to understand the significance they give toward meeting with their family physician. Finally, it is also recommended to conduct a longitudinal study that will examine the perceptions of both patients and family physicians for old age.

## Conclusion

The goal of the current study was to examine the perceptions of 16 family physicians and the differences in their attitudes about men and women’s sexuality in old age. Men were perceived as wanting to engage in full sexual intercourse while among women; there was more of a need for intimacy and a relationship. It was found that most complaints about sexual function in old age come from men experiencing ED. Women seek help less frequently and their direct complaints often relate to gynaecological symptoms, rather than sexual function problems. Family physicians perceived men’s problems as simple, while women’s complaints were perceived as more complex.

## Ethics

The Hospital Ethics Committee approved study No. 0262-16-MMC. The researchers identified the participants and requested their written consent to participate in the study.

## Data and materials

Research data is available upon request.
